# New heat-treated vs electropolished nickel-titanium instruments used in root canal treatment: Influence of autoclave sterilization on surface roughness

**DOI:** 10.1371/journal.pone.0265226

**Published:** 2022-03-18

**Authors:** Rahaf A. Almohareb, Reem Barakat, Fatimah Albohairy

**Affiliations:** 1 Department of Clinical Dental Sciences, College of Dentistry, Princess Nourah bint Abdulrahman University, Riyadh, Saudi Arabia; 2 Department of Clinical Dental Sciences, College of Dentistry, Princess Nourah bint Abdulrahman University, Riyadh, Saudi Arabia; 3 Fatima Albohairy, Electron Microscope Research Unit, Health Sciences Research Center, Princess Nourah bint Abdulrahman University, Riyadh, Saudi Arabia; William & Mary, UNITED STATES

## Abstract

Nickel-titanium (NiTi) instruments used to treat root canal infections are affected by autoclave sterilization in various ways. The aim of this study was to compare the effect of autoclave sterilization on two NiTi rotary instruments that undergo different manufacturing treatments: The electro-polished Race and the heat-treated Race Evo, using scanning electron microscope analysis. In this in-vitro study, Race and Race-Evo instruments were subjected to a number of autoclaving cycles (0, 1, 3, 5, and 10). Scanning electron microscopy images were obtained at 3 mm from the tip of the file at 450x and 1000x magnifications. Surface roughness parameters were measured using ImageJ software. The results showed that autoclave sterilization caused a significant decrease in conventional NiTi Race surface roughness. While in Race Evo, surface roughness increased following the first autoclaving cycle. After 10 autoclaving cycles, surface roughness significantly decreased for both Race and Race Evo files.

## Introduction

The physical and mechanical properties of Nickel-titanium (NiTi) alloy have proven to be an invaluable element in treating root canal infections. NiTi launched the era of rotary engine-driven root canal preparation with instruments (files) that possess increased flexibility and resistance to torsional fracture [1], facilitating the preparation of curved and complicated root canal systems [2].

NiTi rotary files have evolved over the last two decades, introducing different NiTi wires, tapers, and cross-sectional designs [3]. Due to their high cost, NiTi files are often reused [4], with some manufacturers specifying the number of re-uses allowed. For example, Race (FKG, La-Chaux-de-Fonds, Switzerland) NiTi files can be used to treat up to 8 root canals [5].

The Race NiTi rotary file is manufactured from an electropolished conventional NiTi alloy. The file has a triangular cross-section with distinct positive cutting angles [6]. Recently, the Race Evo (FKG Dentaire SA, La Chaux-de-Fonds, Switzerland) NiTi rotary file was introduced. Like the Race file, it boasts the same cross-section with an alternating cutting edge that reduces the screw effect and allows better control of the instrument’s passage within the canal [7]. Its wire, however, received a special heat treatment which increases its flexibility and resistance to cyclic fatigue and fracture.

Files used in root canal treatment come in contact with blood vessels, which classifies them as “critical items” that must be sterilized before first-time use and re-use [8]. Sterilization to exclude all pathogenic species is best accomplished using an autoclave that can kill all bacteria, spores, and viruses [9].

A recent systematic review [10], reported that the surface roughness of NiTi files increases with autoclave sterilization; this increase in roughness correlates with the number of autoclaving cycles the instrument is subjected to. These surface changes may negatively affect their performance in root canal treatment, specifically decreasing resistance to cyclic fatigue and propagating instrument separation [11,12].

Scanning electron microscopy (SEM), atomic force microscopy, and non-contact three-dimensional optical profilometry have all been used in studies to examine the surface roughness of root canal instruments [10,12–14]. SEM is excellent for analyzing surface properties because it allows comprehensive structural analysis by secondary electron imaging. Martins et al. [13], demonstrated that SEM at high magnification would detect most endodontic file defects.

The influence of autoclave sterilization on the surface roughness of NiTi files that undergo different manufacturing treatment; electro-polished conventional NiTi alloy Race and heat-treated NiTi Race Evo, has not been reported in the literature. Therefore, the aim of this study was to use SEM analysis to compare the effect of autoclaving cycles on the surface roughness of Race and Race Evo: two NiTi instruments fabricated by the same manufacturer with identical configuration but different manufacturing treatment.

## Materials and methods

This randomized controlled in vitro study was approved by Princess Nourah Bint Abdulrahman University Institutional Review Board, Riyadh, Saudi Arabia (approval no. 21–0260).

Sample size calculation was conducted using G*Power 3.1 software (Heinrich-Heine-Universität, Düsseldorf, Germany) considering an effect size of 0.85, power of 0.95, and α err of 0.05. Thirty NiTi files with the same tip diameter of 0.25mm and a 0.06% taper were used. They were divided into two equal groups. Group (A) Race files (n = 15) and group (B) Race Evo (n = 15). Each group of files was divided into five subgroups of three files each. One subgroup was designated as the control group and was not sterilized in an autoclave. The other four subgroups were subjected to varying numbers of autoclave sterilization cycles (1, 3, 5, and 10). In each cycle, the files were treated at a temperature of 132°C, with a pressure of 29 psi, for a period of 4 minutes, in a steam sterilizer (Steris Amsco century prevac steam sterilizer, v-148h, United States), followed by a 5-minute drying time, which was according to sterilizer manufacturer’s recommendation for sterilization of endodontic files.

### Scanning Electron Microscopy (SEM) and ImageJ analysis

To unify the imaging area in all tested files, a robber stopper with a single line mark was fixed to the end of the file shaft, after positioning the file latch as a reference in a pre-defined position.

The surface roughness of the files was measured using a Scanning Electron Microscope (JSM-IT500HR) at 3 mm from the tip of the file at 450x and 1000x magnification. Fractures due to cyclic fatigue usually occurred at this location [15]. With an accelerating voltage of 10.0 kv, a high-brightness electron gun produced high-resolution images. An Energy Dispersive X-ray Analyzer (EDX) was used to perform elemental identification and obtain quantitative compositional information related to the two sets of files.

ImageJ software, an open-source science image processing program (version 5.2, LOCI, University of Wisconsin), using the roughness calculation plugin, was used to analyze the images and calculate the roughness average (Ra) and root mean square (Rq) and the average maximum peak to valley height (Rz) values. A rise in these parameters indicated changes in the NiTi files’ vertical surface topography.

### Statistical analysis

Statistical analysis was performed using SPSS (IBM Corp. Released 2013. IBM SPSS Statistics for Windows, Version 22.0. Armonk, NY: IBM Corp). Following descriptive analysis of the data, normality was checked using the Shapiro-Wilks test and, accordingly, multi-factorial analysis of variance, paired and independent t-tests, and one-way ANOVA test followed by the Post hoc Tukey test were used. Statistical significance was set at a p value of ≤ 0.05.

## Results

**Fig 1:**
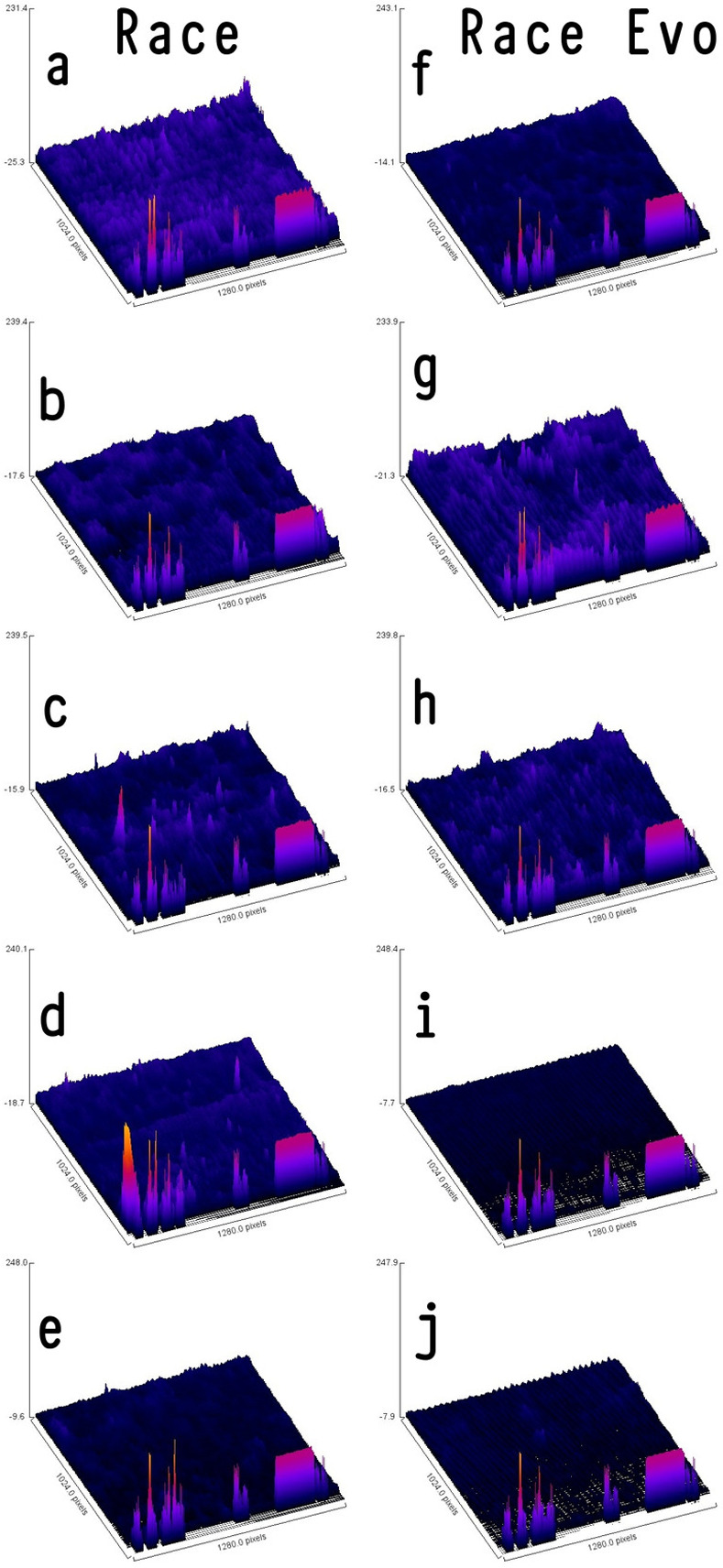
3D color surface roughness profiles of Race and Race Evo files (magnification 450x) where x = 1280 px; y = 1024 px and z (intensity) - 26 to 249 representing the lowest valley and highest peak. a) 0 cycle Race, b) one-time cycled Race, c) three times cycled Race, d) five times cycled Race, e) ten times cycled Race, f) 0 cycle Race Evo, g) one-time cycled Race Evo, h) three times cycled Race Evo, i) five times cycled Race Evo, and j) ten times cycled Race Evo.

Table 1 shows the mean and standard deviation of surface roughness values for Race and Race Evo files after different autoclaving cycles. Before any autoclaving, Race started out with surface roughness parameters higher (Ra = 10.21nm, Rq = 13.32nm, and Rz = 33.14nm) than their newer Race Evo counterparts (Ra = 5.15nm, Rq = 7,20nm and Rz = 25.89nm). However, after the first autoclave cycle, the situation was reversed, with the Race Evo showing higher surface roughness compared to Race. There was a decrease in surface roughness values for both NiTi files associated with 3, 5 and 10 autoclaving cycles (Fig 1).

**Table 1:** Mean and standard deviation of Race and Race Evo surface roughness in different autoclaving cycles.

NiTi	Cycle	Surface Roughness Parameters					
		Rq		Ra		Rz	
		Mean	±Std Deviation	Mean	±Std Deviation	Mean	±Std Deviation
**Race**	0	13.320^a1^	±0.952	10.213^a1^	±0.720	33.140^a1^	±0.257
	1	8.380^b1^	±0.3832	6.183^b1^	±0.303	19.116^b1^	±0.365
	3	8.969^b1^`	±8.969	6.53^bc1^	±1.216	32.704^a1^	±2.868
	5	7.02^bc1^	±0.771	5.004^bc1^	±0.53	26.735^a1^	±4.232
	10	5.492^c1^	±0.256	3.920^c1^	±0.178	16.590^b1^	±3.422
**Race Evo**	0	7.201^a2^	±0.3286	5.154^a2^	±0.306	33.1402^a2^	±0.525
	1	12.014^b2^	±0.189	9.029^b2^	±0.195	42.985^a2^	±11.054
	3	10.233^c1^	±0.304	7.670^c1^	±0.149	32.146^a1^	±11.688
	5	5.532^d2^	±0.427	3.971^d2^	±0.328	14.925^b2^	±1.063
	10	6.082^d2^	±0.195	4.422^d2^	±0.143	16.907^b1^	±1.450

Similar letters indicate non-significant values comparing autoclaving cycles within one group of NiTi files. Similar numbers indicate non-significant values comparing Race and Race Evo files in every autoclaving subgroup. Statistical significance was set at p≤0.05.

**Fig 2:**
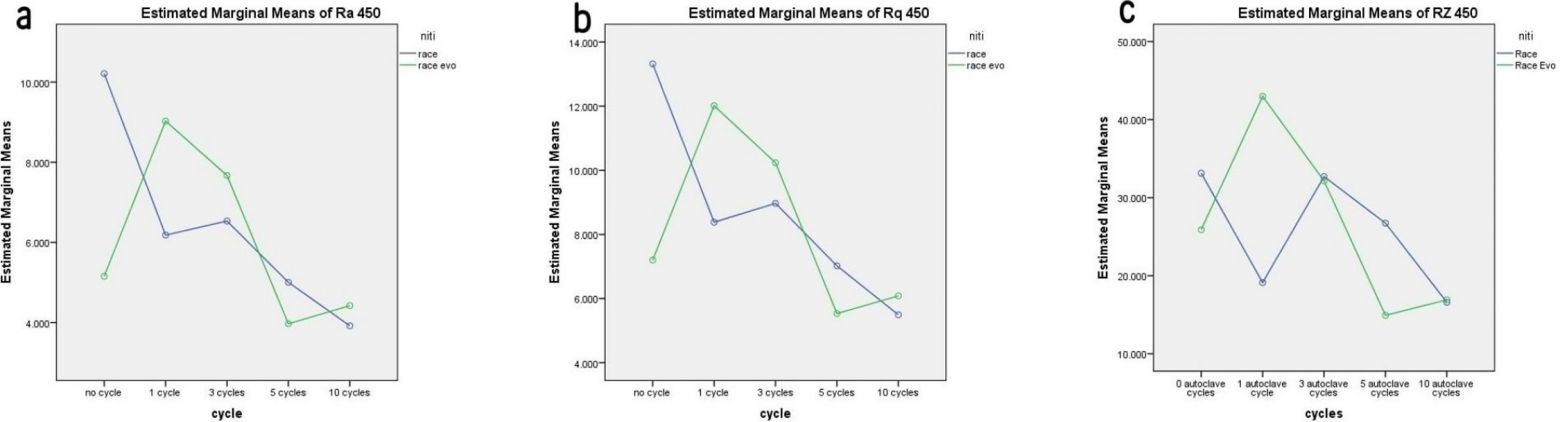
Changes in surface roughness values at 450x magnification according to NiTi file and number of autoclave cycles a) Ra, b) Rq, and c) Rz.

One-way ANOVA followed by Post hoc Tukey test found that surface roughness values for Race Evo significantly differed among all autoclaving cycle subgroups (p ≤ 0.001). For the Race files, however, the surface roughness values (Ra and Rq) were significantly higher in the control group compared to files undergoing autoclaving, whether for 1, 3, 5 or 10 cycles (p ≤ 0.001), while files undergoing 10 autoclaving cycles showed significantly lower surface roughness than all the other autoclaving cycle subgroups (p = 0.005) (Fig 2).

There was no significant difference in surface roughness parameters recorded at 450x magnification and 1000x magnification (Table 2).

**Table 2:** Paired t-test comparison between surface roughness measured at two different magnifications (Rq 450x - Rq 1000x).

Autoclave Cycles	NiTi	Mean	Std deviation	Std error	Sig.
**0**	Race	1.8260	1.550	0.895	0.178
	Race Evo	0.4322	0.254	0.146	0.099
**1**	Race	0.9721	0.698	0.402	0.137
	Race Evo	1.1528	0.973	0.561	0.177
**3**	Race	1.2129	0.948	0.547	0.157
	Race Evo	0.6263	0.143	0.083	0.170
**5**	Race	0.1808	1.425	0.823	0.846
	Race Evo	0.0955	0.151	0.087	0.390
**10**	Race	0.6708	0.315	0.181	0.066
	Race Evo	0.6398	0.445	0.256	0.130

Statistical significance was set at p≤0.05.

Quantitative composition elemental identification revealed an elevated percentage of Titanium and Oxygen in Race Evo (Ti = 43.02±0.15, Ni = 25.84±0.35, O = 30.84±0.13) compared to Race files (Ti = 27.66±0.13, Ni = 22.42±0.37, O = 10.72±0.09).

## Discussion

Sterilization of NiTi files through autoclaving is mandatory in order to minimize the risk of cross-contamination [16]. Many studies have evaluated the effect of autoclaving on the surface roughness of NiTi files, and concluded that surface roughness values increase with the number of autoclaving cycles [17–19]. The failure of NiTi rotary files during clinical use has been associated with the increase in their surface roughness [20,21]. In the present study, however, autoclaving resulted in a decrease in the surface roughness values of Race files. The only time an increase in surface roughness values was recorded, was after the first autoclave cycle of the Race Evo files. After three cycles, the roughness remained higher compared to the control (unautoclaved Race Evo files), but it did not increase compared to the first cycle. These changes were not associated however with alterations in file’s resistance to cyclic fatigue [22].

The discrepancy between this and the previous studies may be related to the use of different assessment methods (SEM vs atomic force microscopy or 3D profiler) and the difference in the autoclaving cycle parameters (temperature/time) [12,14,17,19]. More probably, it may be related to differences in NiTi alloy composition and manufacturing techniques. Van Pham & Vo [23], who used SEM reported a similar decrease in surface roughness values of two heat-treated files: Reciproc Blue and Wave One gold with successive use. While Yilmaz et al. [14], reported that surface roughness values for Hyflex CM files (heat-treated files similar to Race Evo) only increased after 10 autoclaving cycles.

The two most common methods for evaluating the files’ surface roughness are SEM and atomic force microscopy. The issue with SEM is that it can only provide qualitative data. However, recent studies [19,23] have effectively employed image analysis software, which was utilized in this study in order to achieve the quantification of the obtained data.

In accordance with many studies [14,17], the present study showed that the surface roughness of NiTi files made of conventional NiTi alloy was initially greater compared to heat treated files. Nair et al. [17], found that initially, ProTaper conventional files had higher surface roughness, compared to Mwire files (Mtwo). But after the first autoclave cycle, this inversed, with Mtwo files showing increased surface roughness. This was similar to what Race Evo files exhibited in the present study.

No significant changes in surface roughness values with increased magnification were found, which was in contrast to previous studies that reported an increase in surface roughness with increased magnification [14,19]. An explanation for this may be the magnification values used to compare the surfaces: 450x and 1000x magnifications vs 200x and 1000x magnifications in the other studies.

Race NiTi files were reported to have smoother surfaces compared to other conventional NiTi alloy files. This is due to the electro-polishing they undergo, which decreases surface roughness with a controlled electrochemical treatment [24]. As a result, these files possess shiny surfaces with improved mechanical properties that are not altered by chemicals used during canal preparation, such as EDTA and NaOCl [24–26]. This electropolishing also seems to improve adverse surface roughness changes with autoclaving.

Little is known about the manufacturing process of Race Evo files. The manufacturing company states that they undergo heat treatment that triggers a martensite-austenite transition just below body temperature (at 32°-35°C) [5]. The distinct blue color of these files, like the vortex blue, is due to the titanium oxide layer created on the surface by the heat treatment manufacturing technique. This layer improves not only cutting efficiency but resistance to wear [27]. The presence of this layer would explain the higher percentage of Titanium and Oxygen atoms found in Race Evo compared to Race files and may explain the decrease in surface roughness after multiple autoclaving cycles. Reciproc Blue files, which carry the blue color heat treatment layer, showed a similar decrease in surface roughness with autoclaving [23].

An interesting finding in this study was the way the surface roughness of both types of files was altered after the first sterilization cycle. While it decreased significantly in Race files, it increased significantly in Race Evo files. This result indicates that sterilization prior to first use improves the surface quality of Race files, but that is not the case for Race Evo files. It is difficult to explain the current finding due to lack of available knowledge on Race Evo file. A NiTi file’s manufacturing process is instrumental, as NiTi files made from the same alloy, but manufactured using a different machining process (electro discharge machining) behaved differently with autoclaving [14].

The results of this study, along with the reported unaltered resistance to cyclic fatigue with increased autoclaving cycles [22], indicate that both Race and Race Evo files can tolerate multiple autoclave sterilizations without significant alteration affecting their clinical performance.

Finally, a main limitation of this study is the in-vitro setting that does not reproduce the entirety of the complicated factors associated with clinical situations, such as irrigating solutions and dynamic forces the file is subjected to between autoclaving cycles.

## Conclusion

Pre-autoclaving, the surface roughness of the new heated Race Evo files was less compared with the conventional NiTi Race. Autoclave sterilization caused a significant decrease in conventional NiTi Race surface roughness. While in Race Evo, surface roughness increased following the first autoclaving cycle. After 10 autoclaving cycles, surface roughness values significantly decreased for both Race and Race Evo files.

## Supporting information

S1 Table Detailed dataset for all surface roughness parameters for both Race and Race Evo files as seen under two magnifications x450 and x1000. (XLSX)
